# Charles R. Butler, D.D.S., M.D.

**Published:** 1915-01

**Authors:** 


CHARLES R. BUTLER, D.D.S., M.D.—Dr. Charles R. Butler, of Cleveland, Ohio, died December 16, 1914. He was 82 years of age, having been born in Atwater, Ohio, in 1832. He graduated from the Pennsylvania College of Dental Surgery in 1858. He located first in Cleveland for practice in 1854. After graduating in dentistry in 1858 he studied medicine and graduated in 1865 from the Western Reserve Medical College. He was at one time a partner of Dr. Wm. H. Atkinson. He was a most skillful operator and had a genius for making steel instruments. Some of the best forms of plugger points are the result of his skillful manipulation. He was one of the strong men in the early periods of the profession's building, and did great work not only in Ohio but in the national societies. He practiced nearly sixty years in Cleveland, and was greatly beloved by the profession of his own city as well as by all who knew him. The profession is greatly indebted to him for many mechanical appliances, particularly in the operation field and for much work in professional building in which he was a constant and active worker. He has held many offices of honor in the national, and his own state dental societies, and always had the highest professional aims and ideals uppermost in his efforts to promote the standard of an ethical profession.

His side interests were Masonry and fine jewels. He was one of the few 33rd Degree Masons of the Country and was widely known for his work in this fraternity. He loved fine jewels, diamonds, etc., and was an expert in estimating their values, and was often consulted in regard to them. He was a fine citizen and a useful man in every walk of life that he entered. He had hosts of friends everywhere, almost, who will learn of his death with great regret. His gentle and loving sympathy has helped many a struggling young dentist to keep to the right path, and he was always interested in the young practitioner. We have known him for nearly thirty years and we recall many pleasant hours spent with him in friendly intercourse where he never failed to leave us with the impression that he not only cared for us fraternally but that he had a personal interest as well. It was this characteristic that made him beloved by everyone, and also made him a most helpful friend. It is fine to have passed through life and to leave behind such a rich and satisfactory memory. Dr. Butler's wife remains to mourn his personal loss, in which many, many dear friends will sympathetically join her.

